# The Science-Based Pathways to Understanding False Confessions and Wrongful Convictions

**DOI:** 10.3389/fpsyg.2021.633936

**Published:** 2021-02-22

**Authors:** Gisli H. Gudjonsson

**Affiliations:** ^1^King's College London, Institute of Psychiatry, Psychology and Neuroscience, London, United Kingdom; ^2^Department of Psychology, Reykjavik University, Reykjavík, Iceland

**Keywords:** false confessions, memory distrust syndrome, science-based pathways, wrongful convictions, case studies, experimental studies, community studies

## Abstract

This review shows that there is now a solid scientific evidence base for the “expert” evaluation of disputed confession cases in judicial proceedings. Real-life cases have driven the science by stimulating research into “coercive” police questioning techniques, psychological vulnerabilities to false confession, and the development and validation of psychometric tests of interrogative suggestibility and compliance. Mandatory electronic recording of police interviews has helped with identifying the situational and personal “risk factors” involved in false confessions and how these interact. It is the combination of a detailed evaluation and analysis of real-life cases, experimental work, and community (and prison/police station) studies that have greatly advanced the science over the past 40 years. In this review, the story of the development of the science during this “golden era” is told through the three established error pathways to false confessions and wrongful convictions: misclassification, coercion, and contamination. A case study of a major miscarriage of justice is used to highlight the key issues at each stage of the error pathways and it shows the continued resistance of the judiciary to admit mistakes and learn from them. Science is a powerful platform from which to educate the police and the judiciary.

## Introduction

Gudjonsson (2018) has argued there is currently a solid scientific evidence base for understanding the processes involved in cases of false confession, identifying risk factors, and the evaluation of real-life cases for judicial purposes. The growing evidence base over the past 40 years has been driven largely by in-depth analysis of real-life cases of false confession [either proven or probable] (Gudjonsson, 1992, 2003a; Leo and Ofshe, 1998; Drizin and Leo, 2004; Garrett, 2011).

### Early Developments

Münsterberg (1908) laid the foundation for conceptualizing [the] three different types of false confession (Gudjonsson, 2018), but it was not until the 1980s that further tangible theoretical developments took place. Kassin and Wrightsman (1985), through a rigorous literature review, articulated the three psychological types of false confessions which Münsterberg (1908) had crudely outlined. They labeled them *voluntary* (i.e., not police induced), *coerced-compliant* (i.e., the result of not being able to cope with the custodial/interrogative pressure and merely agreeing with police), and *coerced-internalized* (i.e., police persuade suspects that they committed the crime of which they are genuinely innocent) types. Despite some criticism and suggested refinements (Ofshe and Leo, 1997; McCann, 1998), the three-type classification has withstood the passage of time (Gudjonsson, 2018).

As a part of the early development, Gudjonsson and MacKeith (1982) introduced the term “*memory distrust syndrome*” to describe the memory vulnerabilities and processes that produced internalized false confessions. Numerous case studies (Gudjonsson, 2003a, 2018), as well as experimental evidence (Kassin and Kiechel, 1996; Horselenberg et al., 2003; Van Bergen et al., 2008, 2009, 2010), have supported the crucial role of *memory distrust* in some cases of false confessions. What initially drove the resurgence of scientific interest in false confessions in the 1980s were two cases of miscarriage of justice in the 1970s, one in the USA and another in the UK. They set the scene for a better understanding of the vulnerabilities of young people when manipulated by the police to extract a confession. In both cases the confessions turned out to be false and police coerced.

The case of 18-year-old Peter Reilly in the USA (Connery, 1977), provided an important model for understanding coerced-internalized false confessions (Kassin and Wrightsman, 1985). The other case involved the wrongful conviction of three innocent young persons in London who were convicted of murdering Maxwell Confait (Price and Caplan, 1977). The Confait case led to the establishment of the Royal Commission on Criminal Procedure in 1978 and was followed by the implementation of the Police and Criminal Evidence Act (PACE) and its Codes of Practice in January 1986.

Code C provided improved protection of suspects detained for questioning, which included more rigorous police interviewing procedures (e.g., stipulated length of interviews, breaks, and electronic recording of all interviews), free access to legal advice, and the provision of an “appropriate adult” in cases of young persons (i.e., age <18) and those mentally vulnerable (Gudjonsson, 2003a, 2016). Young age is a well-recognized vulnerability to false confession (e.g., Drizin and Leo, 2004), requiring special procedural safeguards (Panzavolta et al., 2015).

The two 1970s cases of proven false confession created an excellent platform for the development of science-based confession research. The early work in the USA focused primarily on theory development (Kassin and Wrightsman, 1985) and on coercive police tactics (Leo, 1996, 2008; Leo and Ofshe, 1998). In contrast, UK developments focused on psychological vulnerabilities and the development of psychometric tests to assess these vulnerabilities, such as suggestibility and compliance (Gudjonsson, 1992, 1997, 2003a; Gudjonsson et al., 1993). In his review of forensic psychology in the UK, Blackburn (1996) identified the development and validation of the Gudjonsson Suggestibility Scales (GSS1 and GSS 2; Gudjonsson, 1983, 1984, 1987) as an exceptional contribution to forensic psychology. The GSS has been influential in helping to overturn wrongful convictions in the UK (Gudjonsson, 2010) and internationally (Gudjonsson, 2003a).

### Case Studies, Experimental Research, and Community Studies

Gudjonsson and colleagues conducted studies among prisoners and suspects at police stations who reported a history of false confession as well as surveys among community samples (for a review see Gudjonsson, 2018, Tables 5.3, 5.4). The studies show that about 20% of Icelandic prisoners and “regular” suspects questioned at police stations report a history of false confession, with a rate of 33.4% reported among Scottish prisoners (Gudjonsson et al., 2019). A false confession rate of 13.8% was found among 2,726 pupils (mean age = 15.5 years) who had been interrogated at police stations in seven European countries (Iceland, Norway, Finland, Bulgaria, Lithuania, Latvia, and Russia) (Gudjonsson et al., 2009).

Redlich et al. (2010, 2011) conducted similar research in the USA. The findings complement the findings from individual case studies (Gudjonsson, 2018). Laboratory studies have also furthered our understanding of the processes and mechanisms involved in false confession (Clare and Gudjonsson, 1995; Meissner et al., 2010). It is the combination of *real-life case studies* of false confession, *experimental research* emerging in the 1990s, and *community studies* that have advanced the scientific basis of the psychology of false confessions. This knowledge base has been consolidated by the collaborative work of researchers in the USA and the UK (Kassin and Gudjonsson, 2004; Kassin et al., 2010; Lassiter and Meissner, 2010).

The knowledge gained from case studies greatly improved the methodology for evaluating cases, including the development, validation, and application of psychometric tests (Gudjonsson and Gunn, 1982; Gudjonsson and MacKeith, 1988; Wrightsman and Kassin, 1993; Gudjonsson, 1997, 2003a, 2018; DeClue, 2005; Davis and Leo, 2013). The case of Engin Raghip, one of the “Tottenham Three,” was the first in the UK to demonstrate the powerful interplay between a real-life case and scientific [psychological] developments (Gudjonsson, 2003a, 2018). The key development was to provide a scientific explanation for Raghip's “average” pre-trial [1987] suggestibility [GSS 1] scores. When tested again in 1988 by another expert [Gudjonsson], Raghip was abnormally suggestible on two separate tests [GSS 1 and GSS 2], administered 11 days apart. His weakness was his inability to cope with interrogative pressure and this was pertinent to his appeal application.

The Court of Appeal dismissed Raghip's appeal application in December 1988 because of the discrepancy between the pre-trial and post-trial suggestibility scores. This appeared to be the end of Raghip's hope for justice, until Gudjonsson decided to find a satisfactory scientific explanation for the discrepancy in the two experts' findings regarding suggestibility. He discovered that at the time of the pre-trial assessment Raghip had been very suspicious of the defense expert who had apparently annoyed him. This was confirmed by the pre-trial expert. This led Gudjonsson to conduct research into the relationship between suggestibility and suspiciousness and anger, guided by a sound theoretical framework (Gudjonsson and Clark, 1986). He published a scientific paper on his findings (Gudjonsson, 1989), which was crucial to the Home Secretary referring the case again to the Court of Appeal and the successful appeal in December 1991 (Gudjonsson, 2003a, 2018). Raghip was a free man due to the evolving science of of forensic psychology.

### Expert Testimony and Judicial Resistance

A recent survey of experts in the field of confessions (Kassin et al., 2018) has shown a consensus that there is a sufficient evidence base to assist jurors in their evaluation of the reliability of confession evidence. Not only were explicit threats and promises during police interviews seen as risk factors for false confessions, but also *false evidence ploy* and *minimization tactics* that imply leniency by offering sympathy and moral justification. The experts also strongly agreed that the risk during police questioning is more prominent among adolescents, persons with compliant or suggestible personalities, and those with intellectual impairments and other mental health conditions.

Despite the impressive evidence base, there has been police and judicial resistance to the notion that suspects would falsely confess to crimes they did not commit (Gudjonsson, 2003a; Kassin, 2014), but innovations in DNA technology have proven that many defendants have in fact been wrongfully convicted based on false confession evidence (Scheck et al., 2000; Garrett, 2011; Norris, 2017).

The most fundamental problem with evaluating the nature of police interviews is that the interviews are not always electronically recorded (Gudjonsson, 2003a; Lassiter et al., 2010). This makes it difficult if not impossible to substantiate suspects' claims of coercion, even if true (Gudjonsson, 2018). In the UK, the Police and Criminal Evidence Act [1984], known as “PACE”], makes it mandatory to record electronically all suspect interviews, which has helped to identify coercion and false confessions (Gudjonsson, 2003a, 2018).

### Review Focus and Objectives

This review will show that once false confessions are obtained, they contaminate the entire judicial process from police investigation to prosecution, courts, and appeals. Garrett (2011) notes that one of the lessons from DNA exoneration cases is that “once central evidence is contaminated at the earliest stages of a case, the damage cannot be easily discovered or reversed” (p. 272). The pathways to false confessions and wrongful convictions are often caused by a systemic failure across the entire criminal justice system and a ferocious battle of the establishment to protect the police and the judiciary from criticism and responsibility for their mistakes by placing the blame on the wrongfully convicted persons (Sekar, 2012; Norris, 2017). The author will discuss the science-based pathways to false confessions and miscarriage of justice through Leo and Drizin's (2010) three primary errors: “*misclassification*,” “*coercion*,” and “*contamination*” whilst being cognizant of the recent five-stage cumulative-disadvantage framework recently proposed by Scherr et al. (2020).

Scherr et al. (2020) provide a comprehensive science-based review of how innocent suspects may be subjected to “cumulative disadvantage” through the combined actions of police officers, forensic scientists, prosecutors, defense lawyers, judges, jurors, and appeal courts. This multistage (gestalt) approach provides an excellent psychological framework for understanding how innocence can lead to false confession and wrongful conviction at different stages of the process. Scherr et al.'s (2020) cumulative-disadvantage framework comprises five distinct process stages: Stage 1—“*Precustodial Interviews*;” Stage 2—“*Custodial Interrogation*;” Stage 3—“*Ensuing Investigations*;” Stage 4—“*Guilty Pleas and Trial Convictions*;” and Stage 5—“*Postconviction, Appeals, Exonerations, and Beyond*” (Figure 1, p. 355).

## The Gudmundur and Geirfinnur Cases

Throughout this review, pertinent examples of the three erroneous pathways are provided from a major miscarriage of justice of six convicted persons in Iceland. They are known as the *Gudmundur* and *Geirfinnur* cases and have featured in BBC radio programmes,^1^ the BBC Storyville and Netflix documentary “Out of Thin Air,” and four books (Latham and Gudjonsson, 2016; Adeane, 2018; Cox, 2018; Gudjonsson, 2018).

Table 1 places the coercive treatment of the six convicted persons (i.e., days in solitary confinement, hours of police questioning, and number of police contrived suspects' “face-to-face” confrontations) in historical and international context. The author is not aware of any comparable criminal cases internationally. For examples, detailed leading reviews of DNA exoneration cases in the USA (Drizin and Leo, 2004; Garrett, 2011) appear “mild” in comparison with the Icelandic cases.

**Table 1:** The days in solitary confinement, hours of police interviews, and number of “face- to-face suspects” confrontation.

**Name of suspect**	**Days in solitary confinement**	Interviews (hours)^*^	“Face-to-face” confrontation^**^
Sævar Ciesielski (SC)	741	340	20
Kristján Vidarsson (KV)	682	215	18
Tryggvi Leifsson (TL)	627	124	16
Erla Bolladóttir (EB)	241	120	11
Gudjón Skarphedinsson (GS)	412	160	5
Albert Skaftason (AS)	88	17	16

* *These figures are conservative because many interviews went unrecorded (e.g., EB was taken to Sídumúli Prison on three occasions for a total of about 11 h before her first recorded interview in the Geirfinnur case on 23 January 1976—all unrecorded)*.

** *The investigators arranged suspects' “confrontations” to improve consistency in their statements and used it as by proxy police pressure to obtain confessions (Gudjonsson, 2018)*.

The length of questioning is strongly associated with the rate of false confession (Gudjonsson, 2003a; Drizin and Leo, 2004; Perske, 2008; Kassin, 2014). Kassin et al. (2007) found in a survey of US police officers that the estimated length of interrogation in criminal cases ranged between 1.60 and 4.21 h, in contrast to proven cases of false confession with an average of 16.3 h of questioning (Drizin and Leo, 2004). Pearse and Gudjonsson (1996) found that out of 161 police interviews in England, 95% lasted <30 min (mean = 22 min, range = 2–109 min). In a study of 20 serious criminal cases involving initial denials, followed by coercive interviews and a confession subsequently disputed (Pearse and Gudjonsson, 2003), the average interview time was 2 h and 16 min (range = 22 min−12 h and 42 min). The longest questioning time (almost 13 h) was found in the case of Stephen Miller [one of the “Cardiff Three”], discussed later in this article.

Table 1 shows that in the Gudmundur and Geirfinnur cases the interrogations lasted between 120 (EB) and 340 (SC) hours, apart from AS where it lasted 17 h. In addition, the police arranged suspects' “face-to-face” confrontations ranged between five (GS) and 20 (SC) sessions.

## The Three Main Error Pathways to False Confession

Leo and Drizin (2010) argue, based on current scientific knowledge, that there are three main sequential error pathways that produce false confessions. These are referred to as “*misclassification error*,” “*coercion error*,” and “*contamination error*”.

### Misclassification Error

Several factors have been identified in the literature as pertinent to misclassification errors during the early stages of the investigation, which can lead to cumulative disadvantage throughout all the different process stages of the case. The six factors reviewed below are: (1) subjective interpretation of deception, (2) bias, (3) offender profiling, (4) flawed scientific evidence, (5) speculative investigative hypotheses, and (6) reliance on prison informants.

#### Subjective Interpretation of Deception

The misclassification error, which is fundamental in police coerced false confessions, involves investigators wrongly focusing on innocent people as suspects. In the USA this commonly occurs because of misleading education or the false belief that officers can reliably tell, through verbal/non-verbal signs, when the person interviewed is “lying,” or they rely on some “gut feeling” or “sixth sense” (Leo, 1996; Vrij et al., 2014). False prejudgement of deception typically leads to *investigator response bias* (i.e., strong guilt presumption, often without tangible evidence; Meissner and Kassin, 2002).

#### Bias and Lack of Open Mindedness

St-Yves (2014) outlines several factors that can lead to bias and lack of openness during investigative interviews. These include: undue weight being placed on first impressions, misleading perception of others (e.g., people see what they expect to see), stereotypical thinking (e.g., that offenders always lie, and police officers always tell the truth), prejudices (e.g., being less likely to give the benefit of the doubt to interviewees with a criminal history), unsubstantiated presumption of guilt, and tunnel vision (i.e., confirmation bias).

#### Offender Profiling

Leo and Drizin (2010) note that “offender profiling” and over reliance on profiles can lead to misclassification error. In the Norwegian Birgitte Tengs case, a Swedish psychiatrist had profiled the victim's cousin as a murder suspect [the cousin had a history of sexual misbehavior at school], which led to him being viewed as a “prime suspect” and to his wrongful conviction (Gudjonsson, 2003a Chapter 23).

#### Flawed Scientific Evidence

There are many documented cases where flawed forensic [“scientific”] evidence has misled the police investigation, resulting in misclassification error (Walker and Stockdale, 1999; Garrett, 2011). This includes the “Birmingham six” (Mansfield, 2009) and the “Maguire Seven” (Kee, 1989). Large advances have been made in forensic science investigations over the past 30 years, reducing the likelihood of misclassification error due to this factor (Scheck et al., 2000; Garrett, 2011; Cole, 2012; Gallop, 2019).

#### Speculative [Unfounded] Investigative Hypothesis

Misclassification may be caused by a trivial circumstantial “clue” or highly speculative investigative hypotheses in overzealous investigators and prosecutor (Gudjonsson, 2018). A comprehensive psychological analysis of the statements in the Gudmundur and Geirfinnur cases indicates that the investigators manipulated the vulnerabilities of a young woman [Erla Bolladóttir- who at the time had an infant daughter, low self-esteem, and a compliant personality] to falsely implicate people of interest to police [linked to Klúbburinn—see Boxes 1, 2] in their relentless pursuit of “solving” the Geirfinnur case (Gudjonsson, 2018, Chapter 14).

Box 1 **The Gudmundur and Geirfinnur cases in a nutshell.**

The Gudmundur and Geirfinnur cases involved the disappearance of two unrelated men on 27 January and 19 November 1974, respectively. Gudmundur's disappearance was not viewed as suspicious at the time. In contrast, the Keflavik's Sheriff's Department viewed Geirfinnur's disappearance as suspicious and linked it to smuggling of alcohol, apparently without any investigative foundation. Soon after the Keflavik investigation started unfounded rumors began to spread that two men linked to a popular Reykjavík Club (“*Klúbburinn*”) were responsible for Geirfinnur's disappearance.

No suspects were officially identified and the investigation was closed within a few months, but suddenly officially reopened by the Reykjavík police in January 1976, with deleterious consequences for fairness and justice. The key mistake appears to have been the Reykjavík investigators' focus on implicating men associated with *Klúbburinn* in Geirfinnur's disappearance.

In February 1980, Iceland's Supreme Court convicted three men of killing Geirfinnur: Saevar Ciesielski [SC], Kristján Vidarsson [KV], and Gudjón Skarphédinsson [GS]. Erla Bolladóttir [EB], the only female in the case, was convicted of perjury. At the same time, the Iceland Supreme Court convicted SC and KV, along with a third man, Tryggvi Leifsson [TL] of killing Gudmundur. A fourth man, Albert Skaftason [AS] was convicted of interfering with the crime scene. All six convicted persons received prison sentences ranging from 12 months [AS] to 17 years [SC] (Gudjonsson, 2018, Table 9.4, p. 249).

The two cases were investigated jointly and tried together. However, there were no dead bodies, no forensic evidence, or any credible witness statements. The only evidence used against them was their inconsistent and incoherent coerced confessions. The prevailing pervasive guilt presumptive attitude and behavior across the police, prosecution, remand prison, and judiciary led to Iceland's greatest miscarriage of justice, which took 40 years to [largely] correct.

Box 2 **Brief chronology of early events in the Gudmundur and Geirfinnur cases.**

1. Early December 1975: A police informant implicates SC, KV, and TL in the disappearance of Gudmundur.
2. 12–13 December SC and EB are arrested on suspicion of fraud and remanded in custody. After 7 days in custody EB is unexpectedly questioned about Gudmundur's disappearance and she implicates SC and KV, after which she is released from custody. Soon thereafter SC is interviewed and implicates one further person [TL].
3. Toward the end of December, the investigators begin to question EB about SC's possible knowledge about Geirfinnur's disappearance [police kept no records of these interviews]. Subsequently EB was taken as a witness on three separate occasions to a remand prison for a total of 11 h before she gave a formal witness statement on 23 January 1976, implicating four men apparently of interest to police, known as the “Klúbburinn men.” The four Klúbburinn men were subsequently arrested, after both SC and KV had also implicated them in Geirfinnur's disappearance.
4. In May 1976, the Klúbburinn men were released from custody without a charge. The investigators then turned their attention to implicating SC and KV, and later Gudjón Skarphedinsson [GS] in the murder of Geirfinnur.

Erla Bolladóttir was the key focus of police manipulation at the beginning of both cases (i.e., their linchpin), leading to the unwarranted arrest of the four Klúbburinn men in the Geirfinnur case. In the Gudmundur case, whilst in solitary confinement the previous month she had been coerced to implicate her partner SC and his friend KV. SC then implicated two more of his associates in Gudmundur's assumed death [TL and AS].

In the author's evaluation of the Geirfinnur case, Erla Bolladóttir was manipulated to implicate the four Klúbburinn men, two of whom were of primary interest to police, but a link was needed to provide a connection between them and Erla so that her witness statement would be seen as credible. The police misclassification was in relation to the Klúbburinn men, but the investigators apparently needed the young people to implicate them in the disappearance of Geirfinnur to achieve their objectives (i.e., convictions of the Klúbburinn men). The police investigation derailed after the four Klúbburinn suspects were released from custody due to lack of collaborative evidence [they had ready access to their lawyers and none of them confessed] and they were out of the case for good. The police then tried to “save face” by turning EB, SC and KV into primary suspects in Geirfinnur's disappearance, and in addition later charged them with perjury for implicating the four Klúbburinn men.

In October 1976, SC implicated Gudjón Skarphedinsson [GS], his previous teacher and an educated man, in the Geirfinnur case. GS became the much-needed witness in the case driving to the alleged crime scene in Keflavik Harbor. The guilt presumptive investigators persuaded him that he was involved in the case, although he never had any independent memory if it (Gudjonsson, 2017). The two Icelandic cases, investigated at the same time, show the dangers of manipulating vulnerable witnesses to implicate people of interest to police due to a misclassification error.

#### Reliance on Prison Informants

There was no tangible evidence base for the Reykjavík investigation in either the Gudmundur or Geirfinnur case. The investigators' reliance on a self-serving prison informant in the Gudmundur case paved the path to the flawed Geirfinnur investigation. The most serious omission was that the investigators were never fully transparent about the reliance on the informant, merely claiming that information had been brought to their attention (Gudjonsson, 2018). Evidence subsequently emerged to suggest that the prisoner implicated the wrongly convicted persons in exchange for leniency regarding his own offenses (Gudjonsson, 2018, pp. 303–304).

Garrett (2011) and The Justice Project (2007) have shown the important role of prison informants in causing miscarriages of justice, particularly in murder cases, through claiming that they heard the alleged suspect confess, sometimes in graphic detail. Often there is an undisclosed deal with the police regarding “favor” for the prisoner (Garrett, 2011).

### Coercion Error

Once investigators have misclassified the suspect, typically through misguided subjective deception detection and guilt presumption bias, they may use *interrogative* and *custodial* factors to coerce a confession from the suspect, particularly in high profile cases where police are under public and political pressure to solve the case (Leo and Drizin, 2010; Gudjonsson, 2018). The external pressure on police, as well as internally motivated factors (e.g., ambition, cognitive bias, feelings of power and control, and sense of expediency) may lead to coercive interviewing tactics.

Gudjonsson (1992) notes that the nine-step Reid Technique (Inbau et al., 1986), the dominant interrogation model in the USA up to this day, focuses on investigators identifying the suspect's vulnerabilities and playing on them to obtain a confession. This has resulted in many cases of miscarriage of justice (Gudjonsson, 2003a, 2018; Drizin and Leo, 2004; Leo and Drizin, 2010). False confessions are a contributing factor to wrongful convictions in approximately 30% of exonerations in the USA (Scherr et al., 2020).

#### Interviews Involve a Dynamic Process

Gudjonsson (2003a,b) illustrates the interactive nature of police interviews, where there is an interplay between:

- *Context* (e.g., the nature of the crime, severe public and political pressure on police to solve the case, the nature and strength of the evidence against the suspect—see Leahy-Harland and Bull (in press).
- *Situational factors* (e.g., nature and duration of questioning, length, and type of detention).
- *Personal factors* (i.e., age, mental state/health/conditions, physical conditions, history of trauma, developmental or personality disorders, personality traits, such as suggestibility and compliance, and association with delinquent peers).
- *Protective factors* (i.e., the presence of a lawyer, an “appropriate adult” in cases of young persons (<18 years) and those with mental health or other vulnerable conditions.

Kassin and Gudjonsson (2004) and Kassin et al. (2010) reviewed the importance of different types of situational and personal factors during suspect interviews. The two reviews show how the Reid Technique is comprised of two main manipulative tactics: *maximization*' (i.e., emphasizing the strength of the evidence—“*evidence ploy*”—and increase of anxiety associated with continued denials) and *minimization* (i.e., theme development and providing moral excuses for the crime).

Gudjonsson (2018) provides a list of 17 types of vulnerability [“risk factors”] that have been found to be associated with susceptibility to give a false confession. These are presented in Table 2 and are self-explanatory. It involves both *state* and *trait* factors (Davis and Leo, 2013). Gudjonsson (2003a) provides a detailed psychological framework for the evaluation of cases of disputed confessions and the psychometric instruments available for objectively testing psychological vulnerabilities, including cognitive abilities, suggestibility, confabulation, compliance, and mental health problems (e.g., anxiety, depression, trauma symptoms). Vulnerabilities during police interviews continue to be researched in the UK (Farrugia and Gabbert, 2020), the USA (Morgan et al., 2020), and the Netherlands (Geijsen et al., 2018a,b,c).

**Table 2:** “Risk” factors to false confession (Gudjonsson, 2018, pp. 115–116).

1. *Context* (e.g., pressure on police to solve the case, the relationship of the suspect with the victim and other suspects, having responsibility for dependents at the time of interrogation and confinement, undergoing loss or bereavement with regard to the victim). 2. *Interrogation and custodial factors* (i.e., the length and number of interviews, tactics used, the nature and duration of custody/solitary confinement). 3. *Not understanding the police caution/Miranda rights*. 4. *Youth* (typically under 18 years but may be older in real-life cases). 5. *The “mind set” of the suspect*. Innocence itself may be a risk factor when suspects focus primarily on the immediate effect of confessing (e.g., being released from custody) and naively waiving their legal (Miranda) rights, believing that truth and justice will always prevail and their solicitor will sort it out. 6. *Physical and mental health problems*, including mental illness, anxiety, depression, and specific phobias (e.g., claustrophobia). 7. *Developmental disorders* [i.e., intellectual disability, attention deficit hyperactivity disorder (ADHD), autism, literacy problems]. [Fetal alcohol spectrum disorder (FASD) also falls under this heading]. 8. *Lack of access to prescribed medication while in custody*. 9. *A history of suffering from sexual abuse, violence, bullying, and other traumatic life events*. 10. *Delinquent peers*. 11. *Conduct disorder and antisocial personality traits*. 12. *Frequent contact with the police and involvement in delinquency/criminal activity*. 13. *Substance abuse history*. 14. *Alcohol or substance misuse intoxication or withdrawal at the time of the alleged offense or when in custody*. 15. *Personality* (e.g., suggestibility, compliance, acquiescence). 16. *Cognitive abilities* (i.e., low IQ, memory problems). 17. *Absence of support while in custody and during interviews* (e.g., no legal advice, no appropriate adult when one is required, lack of access to a doctor).

#### Which Coercive Interviewing Techniques Break Down Resistance and Denials?

Pearse (1997) and Pearse and Gudjonsson (1999, 2003) used factor analysis to explore the key influential tactics used to break down resistance in cases of disputed/false confessions. In one study (Pearse and Gudjonsson, 1999), 39 separate interviewing tactics from 18 serious criminal cases were factor analyzed (i.e., the tactics used during each 5-min segment of an investigative interview with the suspect) were analyzed. The study revealed three main factors: “Intimidation” [“Maximization”]; “Robust Challenge,” and “Manipulation.” The items that make up the three tactics, which were typically used in combination, are shown in Table 3.

**Table 3:** The three key interrogation tactics that are used to break down resistance in disputed/false confession cases.

**Intimidation**	**Robust challenge**	**Manipulation**
a. Maximize seriousness b. Maximize anxiety c. Use of others d. Experienced officers e. Multiple assertions f. Manipulation of self-esteem g. Manipulation of details h. Use of silences	a. Challenges: lies b. Continued dispute c. Challenges: inconsistencies d. Interruptions	a. Maximize seriousness b. Minimize responsibility c. Inducements d. Suggest scenarios

The three factors in Table 3 provide the main general tactics across a variety of different cases. This methodology has a limitation in that the factor analysis did not separate different types of crimes being investigated. For example, the type of technique and suspect's coerced responses may be different in sex crimes than murder. In sex crimes, soft psychological manipulation, appeal, and soft challenges (e.g., from reported victims), are sometimes used to gently overcome denials and [assumed or real] feelings of shame and disgust (Gudjonsson, 2003a). In murder cases, the challenges of denials are robust, confrontational and may involve the “good cop- bad cop” routine (Pearse and Gudjonsson, 2003), which can be used either simultaneously or alternatively (St-Yves, 2014).

It is also possible, particularly during lengthy interviews, to factor analyze the interrogation tactics and the suspect's responses to the questioning in each case. Pearse (1997) gives a range of cases where this was done, showing the close correspondence between tactics and responses, which was different across cases of arson, robbery, incest, and murder. The tactics and responses were not only determined by the type of crime but also by the context behind the questions.

Pearse and Gudjonsson analyzed the questioning tactics and responses of Stephen Miller, one of the “Cardiff Three” (Pearse and Gudjonsson, 2003; Sekar, 2012; Gudjonsson, 2018). There were 19 tapes of interviews, the most coercive interview represented in Tape 7. Five main interview tactics were used during the 19 interviews, which were highly coercive and laid the foundation for Miller's false confession:

1. *Mr. Nasty factor*. Repeatedly challenging (over 300 times) Mr. Miller's version of events by claiming he is a liar, continued dispute, raised voices, threats, maximizing Miller's anxiety.
2. *Mr. Nice factors*. Low tone and reassurance accompanied by multiple assertions and implying the existence of evidence.
3. *Manipulation factor*. Emphasizing the experience of the officers, manipulating details, minimizing responsibility, and challenging Miller's version of events with information provided by coerced witnesses.
4. *Poor delivery factor*. The use of multiple questions and assertions.
5. *Persistent pressure factors*. Questioning by multiple officers, emphasizing the serious nature of the offense, and use of subtle inducements (e.g., “There might be a nice way around this, mightn't there?”).

During the questioning in Tape 7, Miller responds by repeated angry denials, raised voice, and signs of distress, but begins to accept that at the time of the murder he was “stoned” on drugs causing memory problems (an admission), and the “possibility” that he could have been at the crime scene. Miller was now an easy prey for manipulation and “Mr. Nasty” handed over the questioning to “Mr. Nice.”

#### The Use [Abuse] of Solitary Confinement to Induce Confessions

In the Gudmundur and Geirfinnur cases, the threat and use of solitary confinement of both witnesses and suspects was most extraordinary in modern criminal history. Witnesses were always under the looming threat that they could be placed in solitary confinement for almost unlimited time if they were deemed not to be co-operating with the investigators. According to the convicted persons, witnesses, and court documents, the threat of solitary confinement was of three types:

- Explicitly stated (i.e., direct threat).
- Implied in conversation or during interviewing.
- Stating or implying that existing solitary confinement would be extended unless they co-operated with police.

Their solitary confinement was extended repeatedly, usually commencing with 30 days, and then for increasingly longer periods. Table 1 shows that in the cases of SC, KV and TL the solitary confinement lasted 741, 682, and 627 days, respectively.

Once in solitary confinement, the primary focus of suspects was on giving the investigators the information they wanted to hear to get out of custody (Gudjonsson, 2018). The suspects were doomed whatever they said, leading to feelings of hopelessness and despair. To help them cope with their deteriorating mental state, they were provided with high doses of psychotropic medication, sleeping tablets, and tranquilizers over long periods of time (Gudjonsson, 2018, p. 308).

Inevitably, the investigators could never independently corroborate the “provoked confabulations,” which were often rejected when they did not match the coerced stories of other suspects, with increasing pressure for more information. The defendants' claims of innocence were not believed, they were ferociously challenged, and their harsh treatment in custody continued, and on occasions was increased when the defendants were seen as not co-operating (Gudjonsson, 2018, pp. 306, 365). The obvious “red flag” that should have brought the investigation to a halt in the early stages is that none of the six suspects could inform on the location of the bodies of Gudmundur or Geirfinnur. The investigators were on a rollercoaster path and simply increased the coercion rather than admitting failure.

Threats and excessive use of solitary confinement at Sídumúli Prison in Reykjavík for coercing confessions or co-operation of suspects was a disturbing feature of the Icelandic criminal justice system since the early 1970s (Gudjonsson, 2018). Sídumúli Prison was originally built for the storage and cleaning of vehicles, but the interior was rebuilt with police cells and became a temporary remand center in 1971. It closed in May 1996 (Working Group Report, 2013). One of the problems with legal procedures in Iceland, is that until 1992 “all persons remanded in custody in Iceland were systematically placed in solitary confinement” over extended periods of time (for review see CPT, 1993, Paragraph 60). This allowed investigators, as seen in the Gudmundur and Geirfinnur cases, to use this loophole in the law to coerce confessions (see Box 3). The *European Committee for the Prevention of Torture and Inhumane or Degrading Treatment or Punishment* (CPT, 1993) raised concern over the abuse of solitary confinement in Icelandic prisons and the poor conditions provided. The Report was particularly critical of facilities at Sídumúli Prison, stating that “they [the detainees] were simply stored in the establishment” (Paragraph 84). The Report also noted in Paragraph 14 that several police detainees whom they interviewed “referred to threats to place or maintain them in solitary confinement,” showing that in the early 1990s the police were still using solitary confinement as a threat to achieve their objectives (i.e., co-operation, compliance, and confessions). The deleterious impact of solitary confinement on mental health is well-documented in the literature (Haney, 2018).

Box 3 **The strengths and weaknesses of the Icelandic police law in 1974.**

In many respects, the Icelandic law on police interviewing at the time of the Gudmundur and Geirfinnur investigation [74/1975], dating back to the 1950s, was advanced and ahead of its time. It mandated open mindedness (i.e., on matters that support both guilt and innocence), the requirement to record interviews, and lying to suspects, misleading or confusing them was not permitted (Gudjonsson, 2018, p. 147).

The main weakness was that the law stipulated that suspects could be interrogated for up to 6 h at a time [Section 40—English translation: “It is not permitted to interview a person for more than 6 h at a time and must receive sufficient sleep and rest”].

The vagueness of the term “6 h at a time” allowed investigators to interpret it the way they wanted and abuse the process. In the Gudmundur and Geirfinnur cases at times suspects were interviewed for more than 6 h continuously, but additionally were sometimes questioned for more than 10 h in a day, often with only short breaks after 6 h. The recording of timing of questioning of the suspects was typically not provided as stipulated by law [another breach] but had to be determined from references in the Sídumúli Prison diaries, which undoubtedly underestimated the length of questioning in police favor.

In 1989, the Reykjavík Police published an interview guideline manual (Lögreglan í Reykjavík, 1989). This is a remarkably advanced document. It focuses on improved professionalism, transparency, humanity, and accountability, the four pillars of fairness and justice that were broken in the Gudmundur and Geirfinnur investigation (Gudjonsson, 2018, p. 463). The manual made it clear that the 6 h referred to questioning during each 24-h period. This clarification for investigators was an important step forward in the interests of fairness and justice.

The most important lesson from the Gudmundur and Geirfinnur cases is that: “*good law and codes of practice are of no use if they are not followed and the court ignores the police breaches*” (Gudjonsson, 2018, p. 463). Another lesson is that ambiguity in the law can be abused to create a cumulative disadvantage for suspects. These key lessons are of international relevance irrespective of jurisdiction.

The abuse of solitary confinement was a serious problem regarding witnesses and suspects in the Gudmundur and Geirfinnur cases. More than 40 years on, solitary confinement is apparently still used excessively by the Icelandic police for questioning purposes.^2^ On 16 March 2017, the Supreme Court of Iceland ruled that the Icelandic State had violated a clause in the constitution that bans any sort of torture or humiliation of citizens (Mál nr. 345/2016; Gudjonsson, 2018, p. 463).^3^ A series of studies into the motivation behind false confessions show that avoiding custody is a commonly reported factor in Iceland, but not in the United Kingdom or the USA (for a review of the studies see Gudjonsson, 2018, p. 111).

The interviewing of witnesses and suspects in the Gudmundur and Geirfinnur cases included all the coercive techniques listed in Table 3: *intimidation, robust challenges*, and *manipulation*.

#### The Fallacy of the “Greater Good”

Resistance across the police, prosecution, and judiciary is common in cases of wrongful convictions (Garrett, 2011; Norris, 2017). It often seems that the wrongly convicted persons are made to carry the blame for the miscarriage of justice caused by the authorities (Sekar, 2012; Gudjonsson, 2018). Within the context of the extensive police [and court] breaches in the Gudmundur and Geirfinnur cases, the investigative lawyer [VS] who led the Keflavik investigation in the Geirfinnur case from the Sheriff's department between November 1974 and June 1975, tried to justify the investigators breaking the rules. When he was interviewed by BBC journalist [Simon] Cox (2018, p. 296), VS admitted that the investigators “broke every rule, but when we did, it was a development of the criminal courts” [or, in the interest of “greater good”]. It is this kind of flawed “mind set” and self-justification that causes miscarriages of justice (Garrett, 2011; Appleby and Kassin, 2016). It has no place in the pursuit of fairness and justice (see Boxes 3, 4).

Box 4 **The “Indian” interrogation technique.**

In July 1976, when the case was getting nowhere after being derailed by the release of the “four wrongly accused” in May 1976, the Icelandic Ministry of Justice brought in a recently retired “heavyweight” police commissioner [Karl Schütz] from the German Federal Police (BKA) (Gudjonsson, 2018, Chapter 10). Schütz set up a task force, questioned suspects and witnesses through an interpreter, and appears to have gradually taken over the investigation during a 6-month period (August 1976–January 1977). This was despite his not speaking any Icelandic, not being an Icelandic citizen, and not questioning suspects in accordance with the law. He taught the Icelandic investigators the so-called “Indian” questioning technique, which he claimed had been used effectively in Germany. It was aimed at confusing people and catching them “off guard” as a way of supposedly “getting to the truth.” This was achieved by asking questions out of logical sequence or context, to “catch them.” The use of the “Indian” technique was in serious breach of Icelandic law, but was used by Icelandic investigators, apparently with the approval of the judiciary (Gudjonsson, 2018, p. 262). It was not an internationally recognized or accepted technique. It shows the dangers of using manipulative and unvalidated interrogation techniques.

#### Comparison Between the Reid Technique and the English PEACE Model

In contrast to the Reid Technique, the English PEACE Model [acronym for Preparation and Planning, Engage and Explain, Account and Clarification, Closure, and Evaluation] focuses on openness, transparency and accountability whilst identifying vulnerabilities as a way of ensuring fairness and justice (Gudjonsson and Pearse, 2011). A number of scientists have provided helpful descriptions of the two interview styles typically associated with the UK (PEACE) and the USA (Reid), respectively (Bull et al., 2009; Meissner et al., 2012, 2014; Vrij et al., 2014; Oxburgh et al., 2016; Snook et al., 2016, 2020).

The PEACE Model is comprised of an information-gathering (“truth finding”) style, used to establish rapport with interviewees and the use of open-ended exploratory questions to elicit information and establish evidence of guilt or innocence. In contrast, the accusatorial style contained in the Reid Technique is guilt-presumptive, uses closed, confirmatory questions to elicit confessions.

The guidance to police officers about interviewing suspects has evolved since its implementation in PACE's Codes of Practice in 1986 (Gudjonsson, 2016; Home Office, 2019). Currently, within the context of vulnerabilities, Code C focuses on the need for *special care* during questioning of vulnerable suspects, the importance of considering individual *circumstances*, and the need for c*orroboration* to ensure reliability of the accounts provided. There is recognition that when questioning psychologically vulnerable suspects there may be difficulties with communication, proneness to confusion, suggestibility, acquiescence, and compliance. This current guideline shows the impact of empirical research, development of pertinent assessment instruments, and legal judgments on police practice (Gudjonsson, 2003a, 2010, 2016, 2018).

### Contamination and Confabulation

After the admission, “I did it,” there is usually an account provided of the material event and its context. This is referred to as a “post-admission” narrative, which in association with the interview process that facilitates it, is central to understanding and evaluating the confession evidence (Leo and Ofshe, 1998). The contamination may involve the suspect being overly eager to assist the police when under pressure (Gudjonsson, 1995, 2003b), problems with coping with the interrogative pressure and confinement and telling the police what they want to hear (Sigurdsson and Gudjonsson, 1996a), and not fully understanding their legal rights (Sigurdsson and Gudjonsson, 1996b; Gudjonsson, 2003a).

#### Confabulation

The most serious form of contamination during police questioning is confabulation, which within the context of police questioning has been defined as: “problems in memory processing where people replace gaps in their memory with imaginary experiences that they believe to be true.” Gudjonsson, 2003a, p. 364). Following the work of Berlyne (1972), Kopelman (1987) provided evidence for a distinction between “*spontaneous*” and “*provoked*” confabulation. *Spontaneous* confabulation is rare and may have an organic basis, whereas *provoked confabulation* is a more common extension of normal memory processes to a “weak” memory trace, and is usually temporary (Kopelman, 2010). Kopelman (2010) considers confabulation to be a central part of internalized false confessions, which is supported by a growing scientific evidence base (Gudjonsson, 2003a, 2018). It is typically preceded by a condition Gudjonsson and MacKeith (1982) describe as “*memory distrust syndrome*” (MDS). Gudjonsson (2003a) define MDS as:

“a condition where people develop profound distrust of their memory recollections, as a result of which they are particularly susceptible to relying on external cues and suggestions” (p. 196).

Gudjonson (2018) describes the context (e.g., of existing memory problems or substance misuse) and circumstances (e.g., the interviewer undermines the suspect's confidence in his/her memory) where this may happen in cases of false confession. Schacter (2007, pp. 120–121) applies the Gudjonsson/MacKeith MDS model to the well-publicized case of Peter Reilly from the 1970s in the USA where coercive interviewing led Reilly to distrust and then abandon his own memory of innocence, referred to as failure in the “*distinctiveness heuristic*” (Schacter, 2007) and wrongly incriminating himself in the murder of his mother. Schacter defines *distinctiveness heuristic* as “a rule of thumb that leads people to demand recollections of distinctive details of an experience before they are willing to say that they remember it” (p. 102). According to Schacter (2007), MDS “can develop when it is plausible that one might forget even a violent crime—perhaps when a person is intoxicated, or believes he could have repressed a horrendous event” (p. 121). Gudjonsson (2003a, 2017, 2018) and Gudjonsson et al. (1999) have described cases where a memory distrust syndrome confabulation led to wrongful convictions.

#### Provoked Confabulation Across Suspects and Witnesses in the Same Case

The Gudmundur and Geirfinnur cases, which were investigated together, show provoked confabulation across five suspects and two key prosecution witnesses (Gudjonsson, 2018). This makes the cases of exceptional interest to scientists. Bizarrely, two prosecution witnesses were coerced into purportedly corroborating the defendants' confabulated confessions apparently through a process of being “provoked” to confabulate themselves. In the Gudmundur case, a prosecution witness [GJ] only began to “remember” being at the murder scene after 5 h of heavy confrontation in the Reykjavík Criminal Court by one of the defendants [AS]. This was after leading questioning in court over 2 days. The defendants' lawyers were not invited to this “court proceeding” and could therefore not observe or challenge what was “covertly” going on in the background. GJ's testimony was subsequently used to convict the four defendants in the case [SC, KV, TL, and AS] without defense lawyers ever having an opportunity to cross-examine the witness. At the appeal against their convictions, the Supreme Court placed weight on the witness's apparent “*special knowledge*” about the basement flat where the murder supposedly took place, but it failed to mention that the police had taken GJ to the alleged crime scene prior to his first incriminating statement in Court on 3 May 1977 (Gudjonsson, 2018, p. 315).

In the Geirfinnur case, a prosecution witness [SOH] developed provoked confabulation after being coerced by the investigators to incriminate the defendants. Ten months later, he withdrew his witness statement after having realized that he had not actually witnessed the material event at all. The authorities responded furiously by accusing him of giving false testimony and he was remanded into solitary confinement, undoubtedly to try to persuade him to revert to his confabulated testimony, which he did not (Gudjonsson, 2018, pp. 227, 465–470). Nevertheless, his testimony was used by the Supreme Court to convict the defendants.

At a news conference on 2 February 1977, German ex-BKA Commissioner Karl Schütz announced that SOH had been a reliable and crucial witness in the Geirfinnur case. Later [October 1977] SOH officially retracted his confession. At the appeal in February 1980, the Supreme Court ruled that SOH had been the delivery van driver to Keflavík on the night of Geirfinnur's death. Around the time of the news conference in 1977, the Minister of Justice thanked Karl Schütz by commenting: “The nation has been unburdened of a nightmare” (Gudjonsson, 2018, p. 267). Six months later, the President of Iceland awarded Schütz and five of his colleagues from the BKA [as well as the person who recommended Schütz to the Icelandic Government] with medals of the Icelandic State. This demonstrates the extraordinary political commitment to the case.

#### The Power of Claimed “Special Knowledge”

An analysis of the process and content of the post-admission statement is crucial for evaluating the reliability of the confession (Leo and Ofshe, 1998). Many salient discrepancies from the known facts about the crime may indicate a coerced and/or confabulated confession whereas correct material facts (e.g., unusual features of the crime, accurate description of the crime scene, victim's clothing), particularly when they are not in the public domain or unknown to police, are seen as powerful corroborative [incriminating] evidence, often referred to as “special knowledge” (Gudjonsson, 2003a). Unfortunately, wrongful convictions are commonly found in cases where police and prosecutors have falsely claimed at trial that the *special knowledge* must have come from the defendant (Garrett, 2011; Appleby et al., 2013).

Garrett (2011) found that out of 40 cases of false confession, 38 (95%) involved the investigators claiming that the suspects had volunteered key details “and also claimed they [the investigators] assiduously avoided contaminating the confession by not asking leading questions…” (p. 20). “These false confessions were so persuasive, detailed, and believable that judges repeatedly upheld the convictions during appeals and habeas review. After years passed, these innocent people had no option but to seek DNA testing that finally proved their confession false” (p. 21). The special knowledge in cases of false confession most probably came from the police, either wittingly or unwittingly. The most credible special knowledge comes from cases where the suspects provide details of the crime that the police did not know, such as the murder weapon (Sutton, 2013).

#### The Five-Sequential-Stage Process Steps to Internalized False Confession

Following the essence of the MDS model, Gudjonsson et al. (2014) suggest that confabulation in cases of internalized false confession typically involve a five-sequential-stage process):

- *Trigger* (e.g., an arrest, a conversation with police, leading questioning, undermining confidence in one's memory), which makes the suspect distrust his/her own memory (i.e., MDS).
- *Plausibility* (i.e., whilst in a confused state, the suspect is persuaded that the material event could possibly have happened). The more serious the alleged offense the higher the threshold for acceptance of plausibility (Mazzoni et al., 2001; Horselenberg et al., 2006).
- *Acceptance* (i.e., the suspect accepts that the event may have happened). This occurs when the suspect has abandoned his own belief or memory of innocence after the “*distinctiveness heuristic*” has set in (for detailed case examples, see Gudjonsson, 2018).
- *Memory reconstruction* (i.e., the suspect attempts to make sense of what happened, often aided by police and crime scene visits).
- *Resolution* (i.e., an internalized false confession triggered by police is generally short- lived and once the confused state is resolved, the confession is typically retracted).

#### How Long Does the “Internalization” Last?

In cases of *provoked confabulation*, the internalization tends to be *temporary* (Berlyne, 1972; Kopelman, 1987). Gudjonsson (2003a) concluded that most “internalized” false confessions are resolved within hours, after the suspect is no longer in a confused state, but on occasions they may last months or even years (Gudjonsson, 2018, pp. 458–461). Ofshe and Leo (1997) view the term “internalization” in cases of false confession as misleading because the “police-induced belief change during interrogation is temporary, inherently unstable, and situationally adaptive”…. “Ordinary police interrogation is not sufficient to produce transformative or internalized belief change” (p. 209).

The evidence indicates that in most instances suspects never fully believed or accepted their guilt but confess because at the time, usually due to police pressure, they believed and accepted that it may have or probably did happen (Gudjonsson, 2018). This suggests that the confabulated confessions are not fully “internalized,” even if they are long lasting and are more related to belief change than a stable “false” memory.

There is evidence to support the notion that in most instances the confessions are unstable, temporary, and situationally adaptive, giving credence to Ofshe and Leo (1997) proposition. However, despite their apparent unstable existence, in exceptional circumstances they may have long lasting effects on memory (Gudjonsson, 2003a, Chapter 23; Gudjonsson et al., 1999). Ofshe and Leo (1997) based their proposition on the use of “ordinary police interrogation.” It does not accurately reflect the deleterious consequences of extreme tactics as those found in the Gudmundur and Geirfinnur cases (see Table 1 and Box 4). There is recent experimental evidence that highly suggestive interviews can generate rich false memories of committing a crime (Shaw and Porter, 2015; Shaw, 2020).

#### Expert Testimony of Disputed [“Coerced”] Confessions

Defense experts are sometimes called to give evidence in court about coercive pressures during police interrogation and risk of false confessions (Gudjonsson, 2003a; Kassin et al., 2018; Marion et al., 2019). Direct comments on the veracity of the confession are generally not allowed (Marion et al., 2019), although this varies markedly across different jurisdictions (Gudjonsson, 2003a). Comments on psychological vulnerabilities are more readily admitted than specific comments about police impropriety (Gudjonsson, 2010).

Kaplan et al. (2020) conducted an important study that showed “social science experts” [also known as “confession experts” in disputed confession cases] were significantly more aware of the interrogation tactics and personal vulnerabilities as risk factors in disputed confessions than laypeople. Significant differences were found on four groups of variables: “Prohibited Tactics” (large effect size), “Maximization” (large effect size), “Minimization” [medium effect size], and “Personal Risk Factors” (medium effect size).

#### How the Gudmundur and Geirfinnur Cases Came to Be Re-opened

Helga Arnardóttir [Icelandic TV reporter] and Kristín Tryggvadóttir [Tryggvi Leifsson's daughter], arrived in England on 1 October 2011 with the surviving three diaries that TL had written between 25 October 1976 and 5 December 1977 whilst in custody in connection with the Gudmundur case. The purpose of their visit was to seek expert advice from Gisli Gudjonsson about the diaries' content. After reviewing the lengthy diaries, Gudjonsson stated during a filmed interview that this was important “fresh” evidence that called for a review of the convictions in both the Gudundur and Geirfinnur cases. The interview was broadcast on Iceland's TV 2 Station 2 days later.

On 7 October 2011, the Icelandic Minister of the Interior set up a *Working Group* [“Starfshópur”] to review the investigation and convictions in the Gudmundur and Geirfinnur cases. It consisted of three lawyers and a professor of psychology (Jon Fridrik Sigurdsson).

The Minister had received a petition from 1,190 individuals requesting a review of the two cases (Gudjonsson, 2018). Gudjonsson was appointed as an expert consultant to the Working Group. He worked closely with Sigurdsson [Professor of Psychology] on the psychological evaluation of the confessions of the six convicted persons. The Working Group itself had no investigative powers.

The Working Group completed its Report on 21 March 2013, raising serious concerns about the police investigation, the reliability of the six convicted persons' confessions, and the fairness of the court proceedings. The comprehensive science-based psychological evaluation by the two psychologists was included in a separate chapter in the Report (Gudjonsson, 2018). The psychological evaluation documented many salient contextual, situational, and personal risk factors that were so profound that the authorities had to act. This time, the Icelandic judiciary was under close international scrutiny.

#### The Icelandic Court Cases Review Commission

Following the publication of the Working Group Report, the Ministry of the Interior laid the legal foundation [Regulation of 27 August 2013] for the creation of the Icelandic Court Cases Review Commission in [“Endurupptökunefnd”—in short “Review Commission”]. This was a landmark step. A special prosecutor (David Björgvinsson) was appointed to review the appeal applications of the six convicted persons [the families of the deceased Tryggvi Leifsson and Sævar Ciesielski led the appeal on their behalf—a special Bill was passed in Parliament in December 2014 to allow this]. Björgvinsson reported back his findings and recommendations to the Review Commission. Björgvinsson supported the appeals regarding the manslaughter convictions of four of the men and Albert Skaftason's conviction for interfering with the crime scene but rejected Erla Bolladóttir's appeal application against the perjury conviction in the Geirfinnur case (Gudjonsson, 2018). Björgvinsson had previously worked for the European Court of Human Rights and played an important role in opening up the cases.

On 28 January 2016, the four members of the Working Group and Gudjonsson testified under oath in the Reykjavík District Court at the request of the special prosecutor on behalf of the Review Commission. Gudjonsson and Sigurdsson were asked about their methodology in the evaluation of the reliability of the confessions and their main findings.

The Review Commission, which had investigative powers, completed its comprehensive report on each of the six convicted persons' merit for appeal. In essence, it followed the recommendations of the special prosecutor. The six lengthy reports [there was a separate report for each applicant] are remarkable for their detail, the synthesis of the complex and voluminous material, and clarity of findings. The Commission relied heavily on the psychological findings in the Working Group Report and on the contemporaneous personal diaries of Gudjón Skarphedinsson and Tryggvi Leifsson whilst in solitary confinement at Sídumúli Prison in 1976 and 1977.

Despite the Review Commission Reports' overall good quality and merits, there were important flaws in the Reports, particularly in relation to the rejection of Erla Bolladóttir's perjury conviction application. In the author's view, the two most serious errors were that the Commission ignored crucial evidence supporting Erla's appeal application (e.g., unrecorded witness interviews, including three in a remand prison setting) and attempts to undermine the weight of the two psychologists' findings by blaming them for a minor error in the Minister of Interior's *Working Group Report*. The error had nothing to do with the psychological evaluation and was not scripted by the psychologists. In addition, the Review Commission misleadingly argued that multiple confessors in the cases indicated the suspects were responsible for the deaths of Gudmundur and Geirfinnur (see Box 5).

Box 5 **Multiple false confessors.**

The Commission argued that there were “strong clues” from the confessions that the convicted persons had been responsible for the death of Gudmundur and Geirfinnur. This conclusion was based on the assumed improbability that so many persons, convicted persons and witnesses, would have confessed to the killings in a similar way (Gudjonsson, 2018, pp. 455–456). The Commission failed to consider the evidence that many cases of false confessions involve more than one false confessor (Gudjonsson, 2003a). Indeed, Drizin and Leo (2004) found in a USA study of 125 proven cases of false confession between 1971 and 2001, that 38 (30.4%) involved more than one false confessor (p. 975). Having multiple suspects increases the risk of false confessions as one suspect's false confession is used to coerce a false confession from another. As more false confessors emerge, the pressure on further suspects and witnesses to give false incriminating statements is markedly increased. This is a good example of a cumulative disadvantage of witnesses and suspects.

#### Supreme Court Outcome

The crucial question after the publication of the Working Group Report was whether the Icelandic judiciary had the courage and motivation to overturn the convictions in the Gudmundur and Geirfinnur cases. The Supreme Court had previously rejected strong appeal applications from SC and EB and there were no signs it would break tradition until the special prosecutor and the Review Commission disclosed their findings under growing international pressure. On 27 September 2018, 3 months after Gudjonsson's (2018) book on the cases was published, the Supreme Court overturned the convictions of the five men associated with the disappearances and assumed deaths of Gudmundur and Geirfinnur.^4^ This was a historic moment, but there was a damaging sting in the tail of the judgment: the Supreme Court failed to provide their own critique of what had gone wrong in the two cases and the lessons learned. This could have provided guidance to police, prosecution, and judiciary to help prevent future cases of miscarriage of justice. This was a lost opportunity for improved fairness and justice, not only in Iceland, but internationally.

The judiciary has still not had the courage to quash the perjury convictions, particularly of EB, who remains left with the blame for this miscarriage of justice (Gudjonsson, 2018). On 30 January 2020, the five men [two involved compensation to the families of the deceased men] were paid substantial compensation for their wrongful convictions.^5^ In contrast, Erla has received no compensation and is having to fund an ongoing legal battle to have, in the first instance, the Review Commission's damaging decision nullified before her perjury conviction can be quashed.

## Conclusions

The following conclusions can be drawn from this review:

1. There have been major scientific advances made over the past 40 years in understanding false confessions and correcting wrongful convictions. This can be described as the “golden era” in research, case studies, and judicial impact.
2. Most of the research has been conducted in the UK, USA, and Iceland. Other jurisdictions need to conduct more of their own research into police interviews, psychological vulnerabilities, and miscarriages of justice. There is a need for a coordinated international approach and collaboration.
3. The science has been driven by real-life criminal cases, leading to the development of psychological theories of different types of false confession, better understanding of the processes involved, innovative psychometric tests of interrogative suggestibility and compliance, comprehensive framework for assessment procedures, identification and validation of salient risk factors, improved police interview practices, and positive impact on judicial procedure and outcome.
4. It has been the combination of a detailed evaluation and analysis of real-life cases, experimental studies, community surveys, and prison [and police station] studies that have primarily advanced the science. Legal judgments, and surveys of police officers, expert witnesses, and potential jurors have also added substantially to the current knowledge of false confessions and wrongful convictions.
5. Cases of wrongful convictions typically involve cumulative disadvantage during a multistage process (Scherr et al., 2020). It is important to understand the cumulative impact of this process on fairness, justice, and defendants' mental health.
6. Not specifically addressed in this review is the long-term psychological damage commonly associated with wrongful convictions (Grounds, 2004; Gudjonsson, 2018). This neglected area requires more research.
7. Despite the scientific advances and improved detection of genuine cases of false confession, there is no room for complacency. DNA exonerations have demonstrated that false confessions sometimes happen in serious criminal cases and that police claims of suspects' unique “special knowledge” of the crime scene are frequently factually wrong and misleading to the court. The current review shows that police investigators need to closely follow the law and legal procedures and interview suspects with an open mind, professionalism, transparency, humanity, and fairness.
8. It is essential that police interview techniques continue to improve to ensure fairness and justice. The evaluation of police interviews, for example, through independent research, and the lessons learned need to fed back into police practice. There remains the risk that police interview training and judicial protection (e.g., access to legal advice and “appropriate adults”) could be compromised by financial considerations. It is easy to revert to bad investigative habits and poor judicial oversight.
9. The lesson from the current science-base is that false confessions are typically caused by a dynamic process that involves a combination of contextual (e.g., the nature of the crime, pressure on police to solve the case), situational (i.e., custodial and interrogative pressure), personal risk (e.g., suggestibility and compliance), and protective (i.e., access to a lawyer and “appropriate adult”) factors. These may act independently, but more commonly interact. It is the salience, severity, and number of risk factors combined that increases the risk of false confession by creating a cumulative disadvantage.
10. Each case needs to be analyzed individually whilst being guided by the available science-based evidence.
11. The main research gaps are in relation to better understanding of the subtle and dynamic interplay between different risk factors and providing science-based probability [algorithm] estimates of the overall risk of false confession in each case.

## Author's Note

The author declares that in the summer of 1976 he was a serving detective with the Reykjavík Criminal Investigation Department and met five of the convicted persons at Sídumúli Prison [Reykjavík] in connection with his research into lie detection for an MSc dissertation [1977] in Clinical Psychology at the University of Surrey, England.

## Author Contributions

The author confirms being the sole contributor of this work and has approved it for publication.

## Conflict of Interest

The author declares that the research was conducted in the absence of any commercial or financial relationships that could be construed as a potential conflict of interest.

## Footnotes

^1^https://www.bbc.co.uk/news/resources/idt-sh/the_reykjavik_confessions

^2^http://www.ruv.is/frett/kastljos-i-kvold-einangrun-mannrettindabrot. This case involved an innocent man who the police were trying to coerce to incriminate himself. He was seen to be uncooperative and spent 11 days in solitary confinement and became distressed and confused.

^3^http://icelandreview.com/news/2017/05/11/time-review-rules-solitary-confinement

^4^https://icelandmonitor.mbl.is/news/news/2018/09/27/all_found_innocent_in_gudmundur_and_geirfinns_case_/

^5^Fréttabladid, January 30, 2020. Adalheidur Ámundadóttir.
